# Incidence of invasive non-typhoidal *Salmonella* in Blantyre, Malawi between January 2011-December 2019

**DOI:** 10.12688/wellcomeopenres.17754.1

**Published:** 2022-04-29

**Authors:** Catherine N. Wilson, Angeziwa Chunga, Clemens Masesa, Brigitte Denis, Niza Silungwe, Sithembile Bilima, Heather Galloway, Melita Gordon, Nicholas A. Feasey

**Affiliations:** 1 Institute of Infection, Veterinary and Ecological Sciences, University of Liverpool, Liverpool, L69 3BX, UK; 2 Malawi-Liverpool Wellcome Research Programme, Kamuzu University of Health Sciences, Blantyre, Malawi; 3 Department of Clinical Sciences, Liverpool School of Tropical Medicine, Liverpool, L3 5QA, UK

**Keywords:** Salmonella, infection, bloodstream, surveillance, antimicrobial resistance

## Abstract

**Background:** The Malawi-Liverpool Wellcome Trust Clinical Research Programme (MLW) has undertaken sentinel surveillance of bloodstream infection and meningitis at Queen Elizabeth Central Hospital (QECH), Blantyre, Malawi for 20 years. Previously, three epidemics of *Salmonella *bloodstream infection have been identified. Here we provide updated surveillance data on invasive non-typhoidal *Salmonella* disease from 2011 – 2019.

**Methods:** Surveillance data describing trends in invasive non-typhoidal *Salmonella* disease and associated antimicrobial susceptibility profiles are presented for the period January 2011 – December 2019.

**Results:** Between January 2011-December 2019, 128,588 blood cultures and 40,769 cerebrospinal fluid cultures were processed at MLW. Overall, 1.00% of these were positive for *S.* Typhimurium, 0.10% for *S.* Enteritidis, and 0.05% positive for other *Salmonella *species. Estimated minimum incidence of invasive non-typhoidal Salmonella (iNTS) disease decreased from 21/100,000 per year in 2011 to 7/100,000 per year in 2019. Over this period, 26 confirmed cases of *Salmonella* meningitis were recorded (88.5% *S.* Typhimurium). Between 2011-2019 there was a substantial decrease in proportion of *S.* Typhimurium (78.5% to 27.7%) and *S. *Enteritidis (31.8% in 2011 to 0%) that were multidrug-resistant. Resistance to fluoroquinolones and third-generation generation cephalosporins (3GC) remained uncommon, however 3GC increased amongst *Salmonella* spp. and *S*. Typhimurium in the latter part of the period.

**Conclusions:** The total number of iNTS bloodstream infections decreased between 2011-2019. Although the number multidrug resistance (MDR) *S.* Typhimurium and *S.* Enteritidis isolates has fallen, the number of MDR isolates of other *Salmonella *spp. has increased, including 3GC isolates.

## Introduction

Non-typhoidal *Salmonella* (NTS) describes all serovariants of *Salmonella enterica* except *Salmonella* Typhi and *Salmonella* Paratyphi A, B and C. In humans, particularly in high-income countries, NTS infection is typically associated with self-limiting enterocolitis and bloodstream or focal infection are far less common ^1^. In sub-Saharan Africa (sSA) however, NTS have become one of the most commonly identified causes of bacterial bloodstream infection over the last 40 years ^2– 4^, causing a clinical syndrome known as invasive NTS (iNTS) disease. It has most recently been estimated that NTS causes 29% cases of bloodstream infection across Africa with a case-fatality rate of 20–25% ^5^. Invasive disease can also result in iNTS meningitis ^6^. In Malawi, NTS were first documented as a major cause of invasive disease in humans in 1998 in a report describing the aetiology of paediatric meningitis ^7^. The serovars most commonly responsible for iNTS disease across sSA in both children and adults are *S.* Typhimurium followed by *S.* Enteritidis and this is true of Malawi ^3^.

iNTS disease peaked in Malawi in 2003 ^3^, before declining in both adults and children. The decline in iNTS disease occurred in parallel with multiple public health interventions, which have led to reductions in the frequency of key risk factors for iNTS disease; malaria, human immunodeficiency virus (HIV) and acute malnutrition ^8, 9^.

Antimicrobial resistance (AMR) of NTS isolates associated with invasive disease is high globally, with reports of 50–75% multidrug resistance (MDR) in iNTS disease in sSA (for iNTS disease, MDR is typically defined as resistance to co-trimoxazole, ampicillin and chloramphenicol) and with emerging resistance to fluoroquinolones and third-generation cephalosporins reported ^10^. Extended spectrum beta-lactamase (ESBL)-producing and fluoroquinolone-resistant Salmonellae have hitherto only occasionally been reported in Malawi ^11^ and the first confirmed ESBL-producing NTS from Blantyre was isolated in 2009 ^11^.

Here, we bring the description of trends in iNTS disease and associated antimicrobial resistance in patients presenting to Queen Elizabeth Central Hospital (QECH) up to date to December 2019. 

## Methods

We present a retrospective analysis of culture confirmed iNTS disease and associated antimicrobial susceptibility patterns identified at Queen Elizabeth Central Hospital (QECH), Blantyre, Malawi between January 2011 and December 2019 inclusive.

### Setting

Malawi is a land-locked country in sub-Saharan Africa. The climate of the country is sub-tropical, with an annual rainy season typically from November-March. The estimated population of the country is 17.5 million according to the 2018 census. Malawi is one of the poorest countries in the world, driven by low productivity in the agricultural sector and the limited number of opportunities for non-agricultural related activities ^12^.

### Study site

QECH is the largest government hospital in the Southern region of Malawi, with an estimated catchment area of approximately 1.3 million people across Blantyre district, which includes Blantyre City and the rural Blantyre District surrounds. The hospital receives referrals from local clinics and regional district hospitals. Approximately 10,000 adult and 30,000 paediatric patients ^9^ are treated there each year. The hospital has 1,000 beds and frequently operates over capacity. Malaria is endemic in the region, and the current estimated HIV prevalence in Malawi is 9.2% ^13^. Recently the HIV infection prevalence amongst adult medical inpatients admitted to QECH has been recorded at approximately 40% ^14^.

Routine culture of blood was instituted by the Malawi-Liverpool Wellcome Research Programme ( MLW) in 1998 and of cerebrospinal fluid (CSF) in 2000. During the study period, blood cultures were routinely obtained from adult patients recording an axillary temperature of >37.5°C or with clinical suspicion of sepsis. Blood cultures were obtained from children with fever admitted to the hospital who were negative for malaria parasites on either a thick blood film or rapid-diagnostic test or who were considered to be critically ill regardless of the result of the malaria diagnostic tests or degree of fever. CSF was routinely collected from all patients where meningitis was clinically suspected.

### Laboratory methods

Diagnostic microbiology facilities were provided by MLW. All isolates were identified using standard diagnostic microbiological techniques ^15^. Automated blood culture was performed using aerobic bottles (adults) or paediatric fastidious anaerobic (PF) bottles (BacT/Alert, bioMérieux, Marcy-L’Etoile, France). CSF samples were cultured at 37°C on sheep blood (Oxoid, Category number: CMOO55) and chocolate agar (Oxoid, Category number: CMOO55) for 48 hours in a carbon dioxide incubator (LEEC incubator; Make/Model: GA 2000/2010). Any Gram negative bacteria detected on examination of the Gram stain were further sub-cultured onto MacConkey (Oxoid, Category number: CM0007) and sheep blood agar, followed by further characterisation using a triple sugar iron (TSI) agar (Oxoid, Category number: CM0277) slant and direct sensitivity testing. Salmonellae were identified either by biochemical profile using API 10S or 20E (bioMérieux) or by a combination of their appearance on TSI agar slant (acid from glucose, no gas, trace or full production of hydrogen sulphide) and a negative urease test (a loopful of presumptive colonies stabbed into the urea agar slope, incubated at 37°C for 4 hours or overnight. (Media turns pink if urease-positive bacteria are present and remained unchanged for a negative result). They were serotyped according to the White-Kauffman-Le Minor scheme by the following antisera: polyvalent O and H, O4, O6, O7, O9, Hd, Hg, Hm and Vi antisera (Pro-Lab Diagnostics). MLW audits contamination rates and subscribes to the UK External Quality Assessment Service and WHO External Quality Assessment schemes.

Antimicrobial susceptibility testing was performed following the disc diffusion method using ampicillin (Oxoid, Category number: CT0003B), chloramphenicol (Oxoid, Category number: CTT0013B), cotrimoxazole (Oxoid, Category number: CT0052), cefpodoxime (Oxoid, Category number: CT1612B ) and ciprofloxacin (Oxoid, Category number: CT0425B) according to the British Society of Antimicrobial Chemotherapy methods and breakpoints until December 2018 and EUCAST thereafter, at which point the ciprofloxacin disc was replaced with a pefloxacin disc-test (Oxoid, Category number: CT0661) ^16, 17^. In November 2021, repeat antimicrobial susceptibility testing following EUCAST methods was carried out using pefloxacin on those *Salmonella* Typhimurium isolates found to be resistant to ciprofloxacin or pefloxacin between 2015–2019, plus 20% of the remaining *Salmonella* Typhimurium isolates detected annually. E-tests were carried out on those isolates which showed resistance to pefloxacin ^18^. A 0.5 McFarland *Salmonella* solution was plated on Mueller Hinton agar (Oxoid, Category number: CM0337) plate. Ciprofloxacin e-test strip (Oxoid, Category number: MA0104D) was appropriately added to the Mueller Hinton agar plate with *Salmonella* and incubated at 37°C for 16 – 20 hours. Diminished ciprofloxacin susceptibility was classified on the basis of a minimum inhibitory concentration (MIC) between 0.06mg/L and 1mg/L ^17^. Isolates were described as ‘fully susceptible’ if susceptible to these 5 antimicrobials and multidrug resistant (MDR) if resistant to ampicillin, cotrimoxazole, and chloramphenicol. Laboratory data were routinely entered by the diagnostic microbiology service using the Prelink (Prelink V3.0.2429.0), laboratory information management system and data were stored on a secure Microsoft SQL Server database.

### Estimated minimum incidence of *Salmonella* blood stream infections (BSI)

Paediatric and adult blood culture and cerebrospinal fluid culture giving a positive culture for NTS result between January 2011 and December 2019 were abstracted. Microsoft Excel version 16.16.27 was used to undertake the analyses. Those isolates with missing metadata were excluded from the relevant analyses. Patient age, sex, year of culture and phenotypic antimicrobial resistance profile linked to each positive NTS culture were also available. The antimicrobial resistance profile of each serovar over the study period was described. Population projections were made using the 2008 and 2018 census to provide a denominator for estimate minimum-incidence calculations ( National Statistics Office, Malawi). Projected annual population growth was estimated by dividing the difference in the population size of Blantyre between 2008–2018 by ten (the number of intervening years) and evenly distributing this across the study nine-year period. Further, mean monthly incidence of *S.* Typhimurium, *S.* Enteritidis and other *S.* species between 2011–2019 was calculated from the data provided. Incidence of iNTS bacteriaemia cases during the study period was then estimated.

### Ethics statement

Analysis of anonymised data generated by the blood culture surveillance service at QECH was approved by the College of Medicine Research Ethics Committee (COMREC) of the University of Malawi, approval number P.06/20/3071, approval date 24 ^th^ November 2020.

## Results

**Figure 1.**
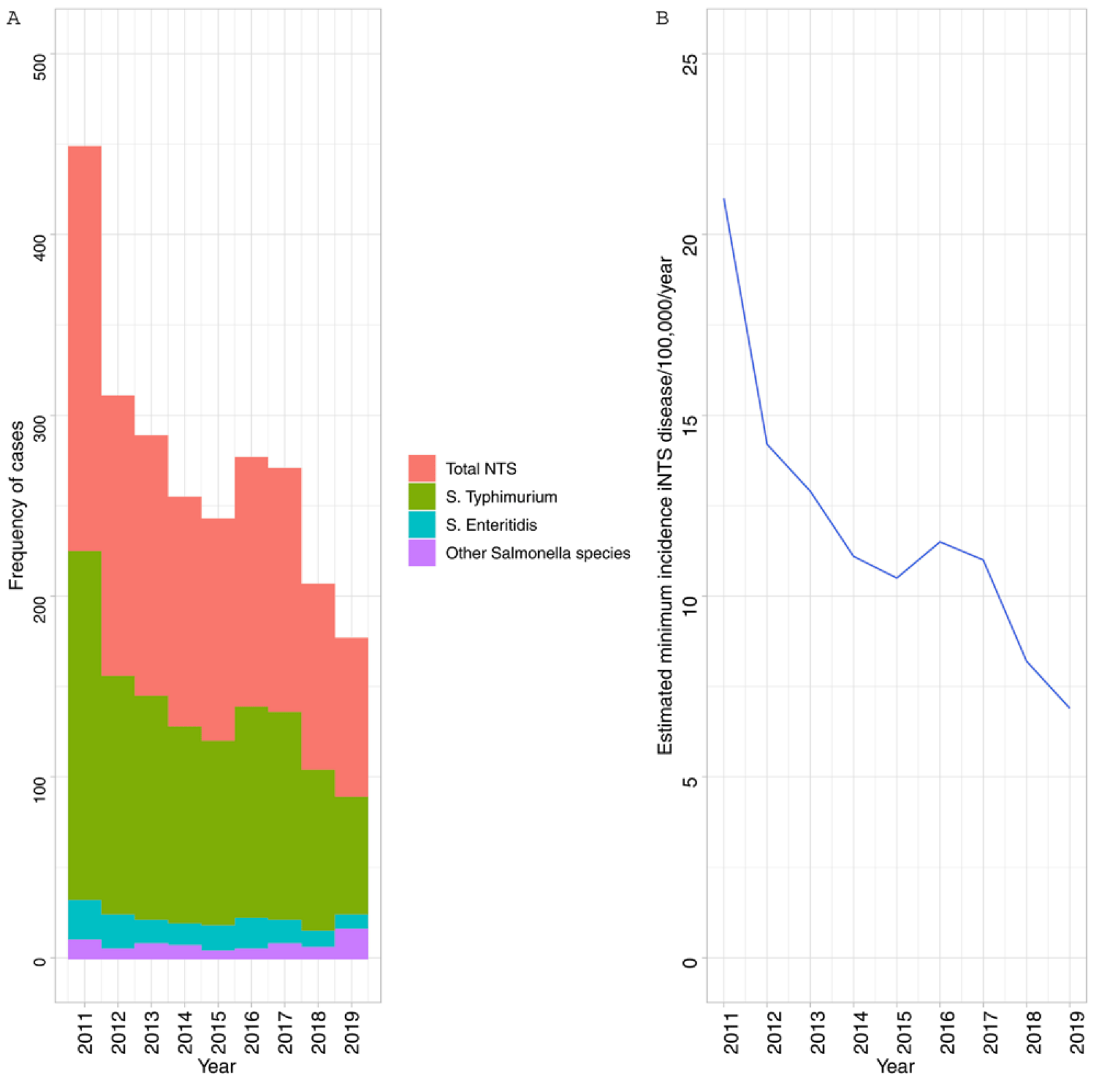
Temporal trends in **A**) Frequency of *Salmonella* Typhimurium, *Salmonella* Enteritidis and *Salmonella* spp. at Queen Elizabeth Central Hospital 2011-2019 and **B**) estimated minimum incidence of invasive non-typhoidal *Salmonella* (NTS) disease in Blantyre.

Between January 2011 to December 2019 128,588 blood cultures were performed at QECH and NTS were isolated from 1,232 (0.96%) ^19^. *S.* Typhimurium represented 84.8 % (n=1,045) of these, *S.* Enteritidis represented 10.3% (n=127) and *Salmonella* spp. 4.9% (n=60). Estimated incidence of iNTS infections declined over the ten-year period from a total of 32/100,000 people/year in 2011 to 11/100,000 people/year in 2019. There were no distinct epidemics of *S.* Typhimurium, *S.* Enteritidis or *Salmonella* spp. and the downwards trend in incidence of iNTS disease continued ( Table 1 and Figure 1).

**Table 1.** Temporal trends in isolation of *Salmonella* and estimated minimum incidence of *Salmonella* bloodstream infection at Queen Elizabeth Central Hospital, Blantyre, Malawi, 2011–2019.

Characteristic	2011	2012	2013	2014	2015	2016	2017	2018	2019
Total Non-Typhoidal *Salmonella*	222	155	144	127	121	138	134	103	88
*Salmonella* Typhimurium	191	132	124	109	104	117	114	89	65
*Salmonella* Enteritidis	22	19	13	12	14	17	13	9	8
Other *Salmonella* species	9	4	7	6	3	4	7	5	15
Estimated Incidence iNTS disease/100,000/y	21.0	14.2	12.9	11.1	10.5	11.5	11.0	8.2	6.9
Estimated population, Blantyre district, thousands	1067	1093	1119	1146	1173	1199	1225	1251	1278

### Monthly temporal trend of invasive non-typhoidal *Salmonella* infection

**Figure 2.**
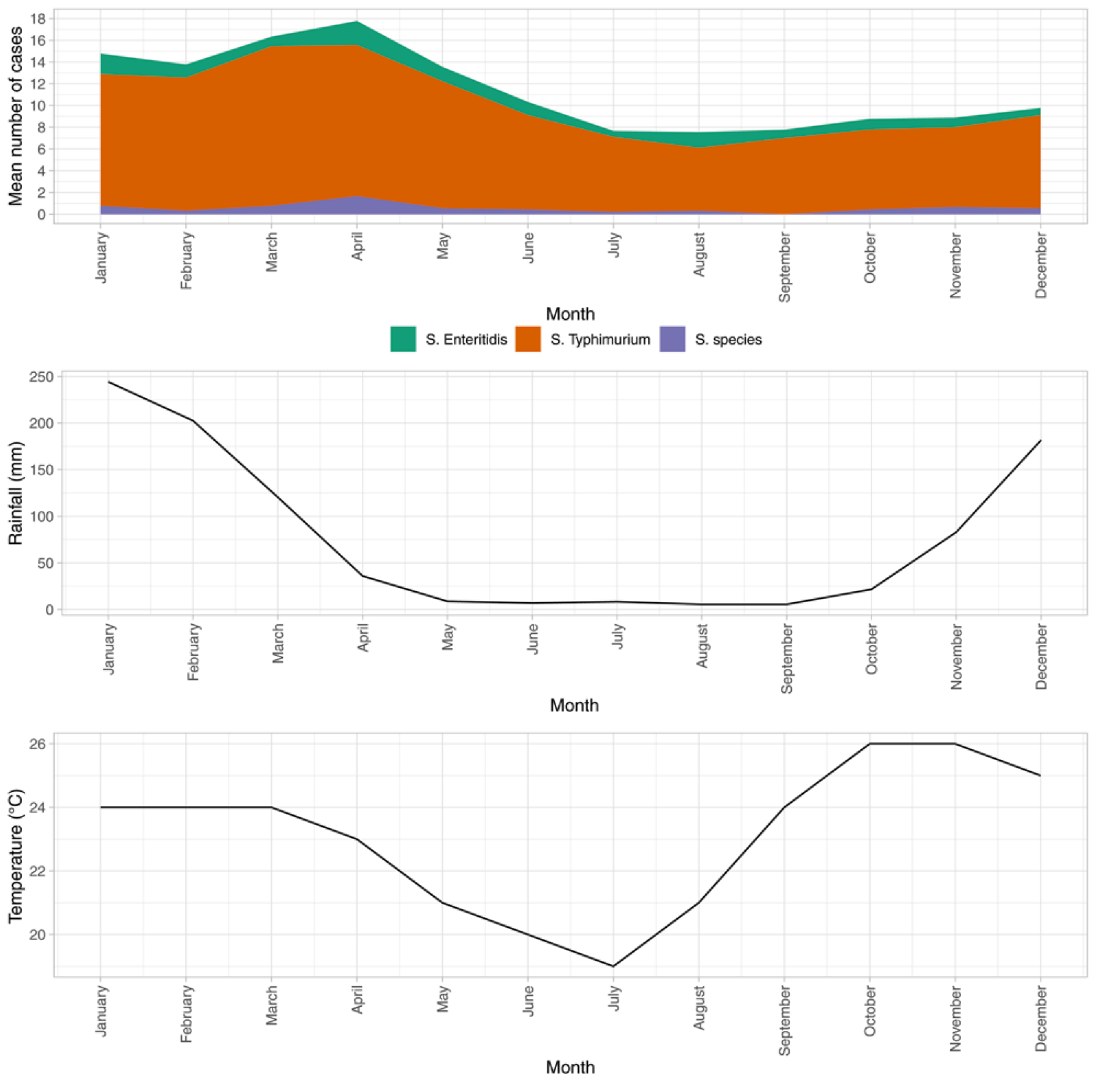
Mean monthly frequency of *Salmonella* Typhimurium, *Salmonella* Enteritidis and *Salmonella* spp. 2011-2019. Temperature and rainfall data from measurements at Chileka International Airport, Blantyre from the Global Surface Summary of the Day database ^21^.

In an analysis of the average number of cases of invasive non-typhoidal *Salmonella* disease per month over the period 2011–2019 we found that the incidence of BSI due to iNTS per month increased from the February rainy season onwards, peaking in April, with a subsequent nadir during the colder and dryer season months of July-September ( Figure 2).

### *Salmonella* meningitis

Between 2011–2019 there were 26 confirmed cases of *Salmonella* meningitis at QECH. The frequency of occurrence was steady over this time period. Overall, 2–6 cases were detected annually, with no clear trend in incidence over the study period. 88.5% (n=23) of these cases were found to be due to *S.* Typhimurium, 3.9% (n=1) due to *S.* Enteritidis and 7.7% (n=2) due to other serovars of non-typhoidal *Salmonella.* The majority of these cases (61.5% (n=16) occurred in children under the age of 18, 75% of whom were under the age of one year old. All of the cases occurring in adults were due to *S.* Typhimurium. Age distribution data can be found as *Extended data* ^20^.

### Antimicrobial Susceptibility

**Figure 3.**
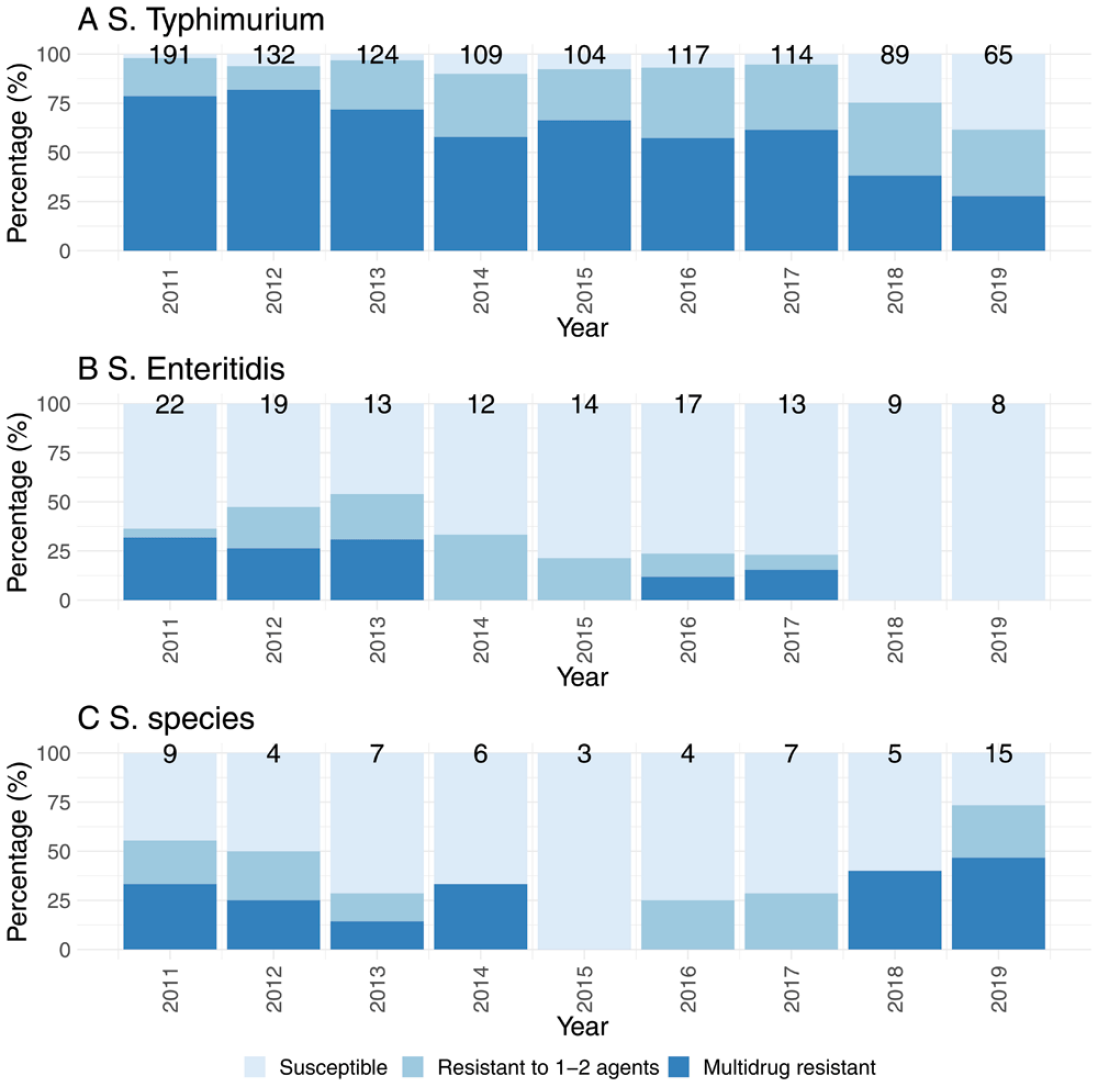
Changes in susceptibility pattern to the first line antimicrobials cotrimoxazole, ampicillin and chloramphenicol for *Salmonella* Typhimurium ( **A**), *Salmonella* Enteritidis ( **B**), *Salmonella* species ( **C**) Number at top of bars indicate the total number of individual isolates recorded during that year.

***Resistance to first-line antibiotics.*** The percentage of *S.* Typhimurium isolates which showed phenotypic MDR to three first-line antimicrobials (cotrimoxazole, ampicillin and chloramphenicol) decreased from 78.5% in 2011 to 27.7% in 2019 ( Figure 3A), predominantly due to re-emergence of chloramphenicol susceptibility. *S.* Enteritidis isolates also showed a similar pattern with a decrease in antimicrobial resistance over the study period ( Figure 3B). This was not due to the re-emergence of susceptibility of *S*. Enteritidis to one particular antibiotic, but rather a disappearance of multidrug resistant isolates. In contrast, whilst there was a decrease in proportion of *Salmonella* spp. isolates that were MDR to three or more antibiotics from within any class during the first three years of the study period, this percentage then increased from 0 to 51.8% (n = 14) between 2017–2019. ( Figure 3C).

***Resistance to fluoroquinolones and third generation cephalosporins (3GC).*** Third-generation cephalosporin and fluoroquinolone resistance remained uncommon, with only 17 ceftriaxone resistant isolates (1.3%, 17/1,284) and 14 fluoroquinolone resistant isolates (1.1%, 14/1,284) across the study period, however we have recently observed the emergence of an ESBL-producing *Salmonella* species that is untypable with locally available antisera. An MDR phenotype was shown by 75% (15/20) of isolates that were resistant to either or both ciprofloxacin and ceftriaxone.

In view of the change in fluoroquinolone susceptibility testing method during the study period, resistance testing was repeated for several isolates. 95 *S.* Typhimurium isolates were retested; 88 previously found to be susceptible by ciprofloxacin disc testing and 7 from across the study period that had been found to be resistant to pefloxacin. Repeat disc diffusion testing revealed 6/95 isolates with disc diffusion inhibition zone diameter indicating resistance to pefloxacin; three from 2019, one from 2017 and two from 2015. E-test results of these six isolates demonstrated two isolates from 2019 with an MIC with intermediate resistance to ciprofloxacin (0.047–0.25µg/ml) and four isolates fully sensitive to ciprofloxacin (<0.06µg/ml). E-tests were also carried out for one *S.* Enteritidis and 11 *S.* species isolates which showed resistance to ciprofloxacin or pefloxacin on disc diffusion testing between 2011–2019. All 12 of these isolates were resistant to ciprofloxacin on e-test, 11/12 fully resistant. Therefore, in total, 20/1,284 isolates (1.6%) showed phenotypic antimicrobial resistance to either or both a third-generation cephalosporin and/or ciprofloxacin ( Table 2).

**Table 2.** Number of non-typhoidal *Salmonella* (NTS) isolates detected displaying phenotypic antimicrobial resistance to a third-generation cephalosporin (ceftriaxone) and fluoroquinolone (ciprofloxacin) antibiotics.

	*S*. Typhimurium (n=5)		*S*. Enteritidis (n=3)		*S*. species (n=12)	
	Ceftriaxone	Ciprofloxacin	Ceftriaxone	Ciprofloxacin	Ceftriaxone	Ciprofloxacin
**2011**	1	0	0	0	0	0
**2012**	0	0	0	0	0	0
**2013**	0	0	0	0	0	0
**2014**	2	0	1	0	0	0
**2015**	0	0	1	1	0	0
**2016**	0	0	0	0	0	0
**2017**	0	0	0	0	1	0
**2018**	0	0	0	0	1	1
**2019**	0	2	0	0	10	10
**Totals**	3	2	2	1	12	11

## Discussion

During the study period 2011–2019, iNTS disease continued to decline in Blantyre, Malawi, however there are three observations of note relating to antimicrobial resistance. Firstly, the decline in MDR *S.* Typhimurium due to re-emergence of chloramphenicol susceptibility; secondly the emergence of a *Salmonella* spp. that is both ESBL producing and ciprofloxacin-resistant; and thirdly findings that ciprofloxacin resistance remains only a sporadic concern for treatment of iNTS disease in Blantyre.

The most marked trend in AMR is the gradual re-emergence of chloramphenicol susceptibility of *S.* Typhimurium isolates, falling from 79% resistance in 2011 to 27.7% in 2019. A similar pattern has previously been shown in *E. coli* and *Klebsiella* spp. isolated at QECH between 1998–2016 ^22, 23^. Use of chloramphenicol as a first-line treatment for sepsis in this setting was superseded by parenteral ceftriaxone and oral ciprofloxacin from 2005, and it is not currently widely used in the community in an oral formulation. Re-emergence of susceptibility to chloramphenicol amongst Salmonellae is an encouraging trend and provides evidence of the potential renewed utility of this previously first-line antimicrobial as a reserve agent for culture confirmed sepsis at QECH where antimicrobial susceptibility is confirmed.

Virtually no MDR *S*. Enteritidis were reported throughout the study period. A large collection of *S*. Enteritidis from the MLW have previously been whole genome sequenced, revealing that MDR isolates belonged to a novel African lineage, whilst fully susceptible ones were part of the global epidemic clade causing enterocolitis in humans, often associated with industrial chicken farming. This raises the possibility that isolates of the global lineage are the predominant cause of *S*. Enteritidis iNTS disease in Blantyre. Further whole genome sequencing of these isolates would confirm this, and if this were the case surveillance of human diarrhoeal illness and investigation of poultry farming practises would be recommended ^24^.

ESBL-producing NTS isolates have previously been extremely infrequently reported in Blantyre ^11^, which is surprising as a diverse range of ESBL genotypes have been reported in other *Enterobacteriaceae* ^25^, and the cephalosporin antibiotic, ceftriaxone, is commonly used in QECH hospital. The emergence of cephalosporin resistance amongst *Salmonella* spp. in the latter part of the study period ( Table 2) is of significant concern and warrants further investigation, particularly as the total number of invasive *Salmonella* spp. detected also increased between 2017–2019 ( Figure 1).

The emergence of *S*. Typhimurium resistant to ciprofloxacin is not yet a cause for significant concern. This serovar is the commonest cause of iNTS disease to be diagnosed at QECH ( Figure 1). Previously, only infrequent ciprofloxacin resistance has been detected in cases of iNTS bloodstream infection in children at QECH ^26^. Ciprofloxacin is a widely available antibiotic in Malawi ^27^. In 2019 the MLW laboratory moved from using a ciprofloxacin disc to infer ciprofloxacin resistance to using a pefloxacin disc, per EUCAST guidance. Pefloxacin is more sensitive at detecting ciprofloxacin resistance, so retesting was carried out on a subset of samples to ensure that fluoroquinolone resistance has not been previously under-reported. Encouragingly, this is not the case. 

## Limitations

These data were captured from a single centre at QECH, Blantyre. Some cases of invasive non-typhoidal *Salmonella* disease will have been missed due to deaths in the community or treatment at local hospital and therefore non-presentation to QECH. Those with drug susceptible disease are more likely to have been treated successfully in the community, making presentation to QECH unnecessary.

## Conclusions

This large longitudinal dataset provides a comprehensive update of trends of invasive non-typhoidal *Salmonella* disease in Blantyre over the period 2011–2019. This emphasises the continual importance of surveillance of bloodstream infections in Malawi, as the decline in iNTS disease identified in the last published survey has continued ^3^. This good news is tempered by the emergence of ESBL producing *Salmonella* spp. which warrants further investigation. The re-emergence of susceptibility of Salmonellae to chloramphenicol provides evidence for the potential renewed utility of this previously first-line antimicrobial as a reserve agent for culture confirmed sepsis at QECH where antimicrobial susceptibility is confirmed.

## Data availability

### Underlying data

Figshare: NTS_QECH. http://doi.org/10.6084/m9.figshare.19294544 ^19^.

### Extended data

Figshare: iNTS_QECH_2011–2019_aggregate_age.docx. http://doi.org/10.6084/m9.figshare.19323668 ^20^.

This project contains the following extended data:

- iNTS_QECH_2011–2019_aggregate_age.docx (aggregate age distribution)

Data are available under the terms of the Creative Commons Zero "No rights reserved" data waiver (CC0 1.0 Public domain dedication).
