# Supplementary material for: Incidence of invasive non-typhoidal Salmonella in Blantyre, Malawi between January 2011-December 2019
Source: Wellcome Open Res. 2022 Apr 29;7:143. [Version 1] doi: 10.12688/wellcomeopenres.17754.1 (PMC10160792; doi:10.12688/wellcomeopenres.17754.1)
Supplement: Peer review history

# Peer review: Reviewer response for version 1

**DOI:** 10.21956/wellcomeopenres.19650.r56252

**Published:** 2023-05-10

**Authors:** Se Eun Park

**Affiliations:** 1 Department of Global Health Security, Yonsei University Graduate School of Public Health, Seoul, South Korea; 2 International Vaccine Institute, Seoul, South Korea

This is an interesting and important manuscript as it presents the up-to-date multi-year iNTS disease epidemiology in Blantyre, Malawi. It provided the latest trend on the annual incidence including seasonality and the AMR/MDR patterns per serovars. Please find below some further thoughts for the authors' consideration:

 Introduction notes the decline in iNTS disease occurred in parallel with multiple public health interventions, which led to reductions in frequency of key risk factors for iNTS disease such as malaria, HIV and acute malnutrition. In the methods, it notes that malaria is endemic in the region and recent HIV infection prevalence in adult medical inpatients admitted to QECH nearly 40%. It seems these two sentences are contradicting, leading to confusion on whether or not we can presumably interpret these underlying risk factors to be a possible contributing factor to the reduction of iNTS disease incidence presented in the results.

 In methods, on the samples tested for AST, it notes ‘plus 20% of the remaining Salmonella Typhimurium isolates detected annually’. Could the authors please elaborate the rationale behind this 20% cut-off/selection algorithm?

 Methods on estimated incidence: 'minimum-incidence' definition? Do authors mean crude incidence (annual)? Or crude incidence person-years-observation (PYO)? It seems, per Table 1, the minimum-incidence is referring to crude annual incidence. It would be good to further clarify the definition of the terminology of minimum-incidence used and its formula.

 Results on resistance to first-line antibiotics: Would it be possible for the authors to look into any potential factors (such as geographic patterns (of patients from whom the samples were obtained), age, pre-treatments/community level use of or practice on antibiotics, etc.) in relation to the increasing trend between 2017-2019 (as the resistance trend has been going down in the previous years). Also, would it be possible to look into what the other *Salmonella* species were and add (for result: Table 2)?

 Discussion: would it be possible for the authors to also discuss about the risk factors mentioned in the introduction, in relation to the iNTS disease incidence trend and AMR/MDR profiles exhibited in the results?

 Source data: I couldn’t download the complete source data via the link provided in the manuscript ‘data availability’ section. Perhaps it is a technical error but it would be good to double check the availability.

Is the work clearly and accurately presented and does it cite the current literature?

Yes

If applicable, is the statistical analysis and its interpretation appropriate?

Yes

Are all the source data underlying the results available to ensure full reproducibility?

Partly

Is the study design appropriate and is the work technically sound?

Yes

Are the conclusions drawn adequately supported by the results?

Partly

Are sufficient details of methods and analysis provided to allow replication by others?

Partly

Reviewer Expertise:

iNTS disease epidemiology in sub-Saharan Africa

I confirm that I have read this submission and believe that I have an appropriate level of expertise to confirm that it is of an acceptable scientific standard.

# Peer review: Reviewer response for version 1

**DOI:** 10.21956/wellcomeopenres.19650.r56257

**Published:** 2023-05-04

**Authors:** Min Yue

**Affiliations:** 1 Institute of Preventive Veterinary Sciences & Department of Veterinary Medicine, Zhejiang University College of Animal Sciences, Hangzhou, China

A brief descriptive study regarding a local surveillance study in a Malawi hospital between January 2011 to December 2019 was conducted. It represented an important update on knowledge of iNTS in sub-Saharan Africa area. I have only a few minor comments that may help to improve the manuscript.

1. The estimated incidence should project with a standard deviation to indicate minimum and maximum, where the latter is missing here.
2. In figure 2, there is a nice curve for the correlation, is there any correlation between no. of case and rainfall, or between no. of case and temp.? Two linear correlation analysis would project such correlations.
3. There is some overlapping information between table 1 and figure 3. I would suggest that table 1 could be made as figures. Similar issue could be conducted with table 2.
4. Concerning the MDR isolates, it would be great to combine the analysis of antibiotics prescription. Is there any correlation between the choice of antimicrobials and MDR isolates?
5. iNTS in elsewhere of the word should be indicated as the comparative purpose. Additionally, the suggested updated antimicrobials for treatment should be stated clearly in the conclusion part.
6. There seem to be some controversial findings when reading ref #26, #27 with increasingly key AMR prevalence. Is there any explanation for that?

Is the work clearly and accurately presented and does it cite the current literature?

Partly

If applicable, is the statistical analysis and its interpretation appropriate?

Yes

Are all the source data underlying the results available to ensure full reproducibility?

Yes

Is the study design appropriate and is the work technically sound?

Yes

Are the conclusions drawn adequately supported by the results?

Yes

Are sufficient details of methods and analysis provided to allow replication by others?

Yes

Reviewer Expertise:

Salmonella, genomic epidemiology, public health, antimicrobial resistance

I confirm that I have read this submission and believe that I have an appropriate level of expertise to confirm that it is of an acceptable scientific standard.

## References

[1] : A nontyphoidal Salmonella serovar domestication accompanying enhanced niche adaptation. *EMBO Mol Med* .2022;14(11) : 10.15252/emmm.202216366 e16366 10.15252/emmm.202216366 36172999PMC9641423

# Peer review: Reviewer response for version 1

**DOI:** 10.21956/wellcomeopenres.19650.r56256

**Published:** 2023-04-28

**Authors:** Robert Kingsley

**Affiliations:** 1 Quadram Institute Bioscience, Norwich, UK

This is an important update on the epidemiology and antimicrobial susceptibility of iNTS disease isolates in Malawi during the period 2011 to 2019. The work carried out at QECH continues to be the most complete analysis of this disease, that is also of significance in many countries in sSA. A decrease in the incidence of the isolation of Salmonella is reported along with phenotypic MDR. Resistance to fluoroquinolone and third generation cephalosporinsI was not considered a current concern for treatment. The emergence in 2019 of an unidentified serotype of Salmonella with resistance to 3GCs is reported. I only have a few minor comments that I hope will improve clarity of this excellent surveillance and analysis.

 Please clarify how Typhimurium and Enteritidis were identified by serology using ‘ *polyvalent O and H, O4, O6, O7, O9, Hd, Hg, Hm and Vi antisera*’ as described. The methodology would appear to be only capable of typing these to serogroup B and D, although I would think these are very likely to be largely Typhimurium and Enteritidis based on previous genomic epidemiology. Figure 3: ‘ *The percentage of S. Typhimurium isolates which showed phenotypic MDR…*’ is interpreted to be ‘ *predominantly due to re-emergence of chloramphenicol susceptibility*’ but this does not seem to be supported by the data. By 2019 the proportion of susceptible isolates is similar or greater than those with resistance to two antibiotics. Furthermore, the increase in susceptible isolates compared to 2011 is much greater over the study period than those with resistance to two.  

 Information about the antimicrobials used to treat febrile infections in QECH and any changes over the study period would be helpful in interpreting the resistance data. Are Ciprofloxacin and 3GCs used as an empirical treatment?  

 Suggest citing Table 2 earlier as I nearly missed this when reading about the susceptibility earlier in the section.  

 For the retesting on perfloxacin, it is not clear if the six found to be resistant were within the seven previously found to be perfloxacin resistant. This final paragraph of the results is difficult to understand and would benefit from editing for clarity.

Is the work clearly and accurately presented and does it cite the current literature?

Yes

If applicable, is the statistical analysis and its interpretation appropriate?

Not applicable

Are all the source data underlying the results available to ensure full reproducibility?

Yes

Is the study design appropriate and is the work technically sound?

Yes

Are the conclusions drawn adequately supported by the results?

Yes

Are sufficient details of methods and analysis provided to allow replication by others?

Yes

Reviewer Expertise:

Microbiology, host-pathogen interactions, Salmonella infection biology, genomics, genomic epidemiology

I confirm that I have read this submission and believe that I have an appropriate level of expertise to confirm that it is of an acceptable scientific standard.
